# Plasma-Enhanced Atomic Layer Deposition of Cobalt Films Using Co(EtCp)_2_ as a Metal Precursor

**DOI:** 10.1186/s11671-019-2913-2

**Published:** 2019-03-04

**Authors:** Bao Zhu, Zi-Jun Ding, Xiaohan Wu, Wen-Jun Liu, David Wei Zhang, Shi-Jin Ding

**Affiliations:** 1 School of Microelectronics, Fudan University, Shanghai, 200433 China; 2 Department of Materials Science, Fudan University, Shanghai, 200433 People’s Republic of China

**Keywords:** Co films, Atomic layer deposition, Low resistivity, Low deposition temperature

## Abstract

For advanced Cu interconnect technology, Co films have been widely investigated to serve as the liner and seed layer replacement because of a better wettability to Cu than Ta. In this article, the Co films are grown by plasma-enhanced atomic layer deposition using Co(EtCp)_2_ as a precursor, and the influences of process parameters on the characteristics of the Co films are elaborately investigated. The results indicate that the process window is 125–225 °C with a growth rate of ~ 0.073 Å/cycle. That is to say, the connection of Et group to Cp ligand can enable a stable film growth at 125 °C, while the corresponding temperature must be higher than 200 °C in terms of Co(Cp)_2_ and Co(MeCp)_2_. The deposited films contain N and O elements besides dominant Co and C. Furthermore, the prolongation of the NH_3_ pulse time significantly enhances the conductivity of the Co film and a low resistivity of 117 μΩ cm can be achieved with a NH_3_ pulse time of 40 s. The root mean square roughness shows a smaller variation with the deposition temperature and maintains a low value of ~ 0.3 nm, indicative of a flat Co film.

## Background

Considering the conventional Cu interconnect process in high-speed ultra-large scale integrated circuits, a barrier layer such as TaN is indispensable to prevent the diffusion of Cu atoms into the surrounding interlayer dielectrics (ILD) [1]. Besides the barrier layer, a liner layer like Ta is also necessary to enhance the adhesion between the barrier layer and Cu. In addition, it is difficult for the Cu line to be deposited on the liner layer by electroplating directly due to the weak nucleation of Cu atoms on the Ta surface. As a consequence, a Cu seed layer is needed to be coated on the liner layer prior to the electroplating of Cu. That is to say, a stack of TaN/Ta/Cu seed layer must be inserted between the ILD and Cu line. Furthermore, this stack is coated on the ILD patterned as trenches and vias. With the downscaling of the device feature size, the volume available for Cu interconnect line steadily decreases. In order to achieve a lower Cu interconnect resistance, seedless barrier/liner layer has been widely investigated [2–6]. For example, TaN still works as the barrier layer and Co replaces Ta as the liner layer. Due to a better wettability of Co to Cu than Ta, Cu can be electroplated on the Co surface directly. Traditionally, the barrier/liner layer is grown by physical vapor deposition process. However, deposition of a high-quality barrier/liner layer is challenging since PVD has a poor step coverage rate in high aspect ratio trenches and vias. Instead, ultrathin, continuous and good step coverage films can be obtained by atomic layer deposition (ALD) technique thanks to the self-limiting growth property [7].

In terms of ALD Co films, a large number of Co precursors, especially the ones based on cyclopentadienyl ligand (Cp), have been widely studied, such as bis-cyclopentadienyl cobalt (CoCp_2_) [8–14], bis(η-methylcyclopentadienyl) cobalt [Co(MeCp)_2_] [15], and cyclopentadienyl isopropyl acetamidinato cobalt [Co(CpAMD)] [16]. The employment of CoCp_2_ enables the growth of Co films with a low resistivity and high purity; however, the stable film growth is limited to the temperatures beyond 250 °C. With the addition of methyl group to the Cp ligand, a lower temperature growth can be achieved at 200 °C, which is attributed to the higher reactivity of NH_3_ radical to the MeCp ligand compared with Cp ligand. On the basis of Co film growth with CoCp_2_ and Co(MeCp)_2_ as precursors, the process window could be moved to lower temperatures, i.e., < 200 °C if ethyl group is connected to the Cp ligand.

In this work, Co thin films were grown by plasma-enhanced ALD (PE-ALD) using bis(ethylcyclopentadienyl) cobalt [Co(EtCp)_2_] and NH_3_ plasma as precursors. The influence of different process parameter on the characteristics of the Co films was elaborately investigated. As a result, a process window of 125–225 °C was achieved successfully. In addition, the Co films exhibit a lower resistivity (~ 130 μΩ cm).

## Methods

Various Co thin films were grown by PE-ALD on a 200 nm SiO_2_ film, which was deposited on p-type silicon substrates by thermal oxidation. Co(EtCp)_2_ was used as the metal precursor, which was stored in a container at 70 °C and transferred into the deposition chamber with a N_2_ carrier gas. The NH_3_ plasma was generated by a remote plasma generator under a power of 2800 W, acting as the reducing agent. The flow rate of N_2_ was kept at 50 sccm, and the working pressure was ~ 1000 Pa during the film growth. To investigate the effect of deposition temperature on the film growth, the substrate temperature was varied from 100 to 270 °C with a step of 25 °C. Moreover, to optimize the process parameters, the pulse times of Co(EtCp)_2_ and NH_3_ plasma were also changed, respectively. In addition, in order to investigate the effect of post annealing on the Co films performance, the samples deposited at different temperatures were annealed in the forming gas (N_2_/4%-H_2_) at 400 °C for 30 min.

The thickness and density of the film were deduced by X-ray reflection, and the microstructure of the film was determined by grazing incidence X-ray diffraction (XRD) on a diffractometer (Bruker D8 Discover) with Cu K_α_ radiation. The surface morphology of the film was observed with atomic force microscopy (AFM) (Bruker Icon) and scanning electron microscope (SEM) (Zeiss SIGAMA HD). The elemental composition and chemical bonds of the film were analyzed by X-ray photoelectron spectroscopy (XPS) (Kratos Axis Ultra DLD). The sheet resistance of the film was measured by four-point-probe, and the film resistivity was calculated based on the film thickness and the sheet resistance.

## Results and Discussion

### Optimization of the ALD Process Parameters

**Fig. 1:**
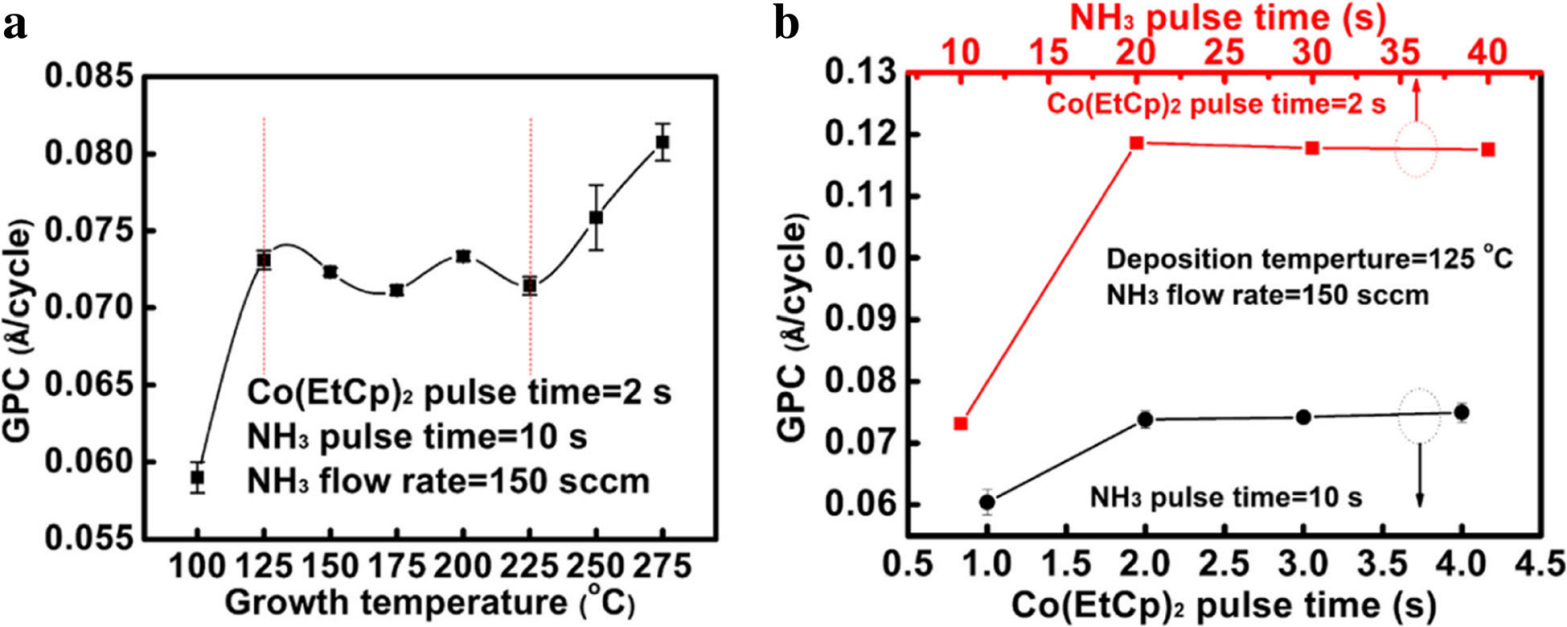
Dependence of the growth rate of the ALD film on **a** substrate temperature, **b** Co(EtCp)_2_, and NH_3_ pulse time

Figure 1a shows the growth rate of the Co film as a function of substrate temperature. It is found that the growth rate increases upon increasing the substrate temperature to 125 °C, and then a relatively stable growth rate of 0.073 ± 0.02 Å/cycle is obtained between 125 and 225 °C. However, when the substrate temperature goes up to 250 °C or higher, the growth rate is increased. Therefore, the temperature range of 125–225 °C can be considered as an appropriate process window. Compared with Co(Cp)_2_ and Co(MeCp)_2_, the addition of Et group to Cp ligand enables the process window moved to a lower deposition temperature, indicating a higher reactivity of EtCp ligands with NH_3_ plasma. Such low temperature growth at 125 °C is beneficial to reducing the thermal budget. Regarding the substrate temperatures lower than 125 °C, the relatively slower film growth should be ascribed to the lack of adequate activation energy for the chemical reaction [7]. Such a high growth rate at ≥ 250 °C is related to thermal decomposition of the metal precursor [17]. To investigate the influence of Co(EtCp)_2_ pulse time on the growth rate of the deposited film, the pulse time of Co(EtCp)_2_ increases gradually from 1 to 4 s with a step of 1 s while other process parameters are fixed. As shown in Fig. 1b, the growth rate increases from ~ 0.06 to ~ 0.073 Å/cycle with the increment of Co(EtCp)_2_ pulse time from 1 to 2 s, and then maintains a relatively stable value. This indicates that the surface adsorption of Co(EtCp)_2_ attains a saturation at a pulse time of 2 s. Figure 1b also illustrates the influence of NH_3_-plasma pulse time on the growth rate of the film. The growth rate increases with prolonging NH_3_-plasma pulse time; however, when the plasma pulse time exceeds 20 s, the growth rate reaches a saturation value of ~ 0.12 Å/cycle.

### Characterization of the Deposited Co Films

**Fig. 2:**
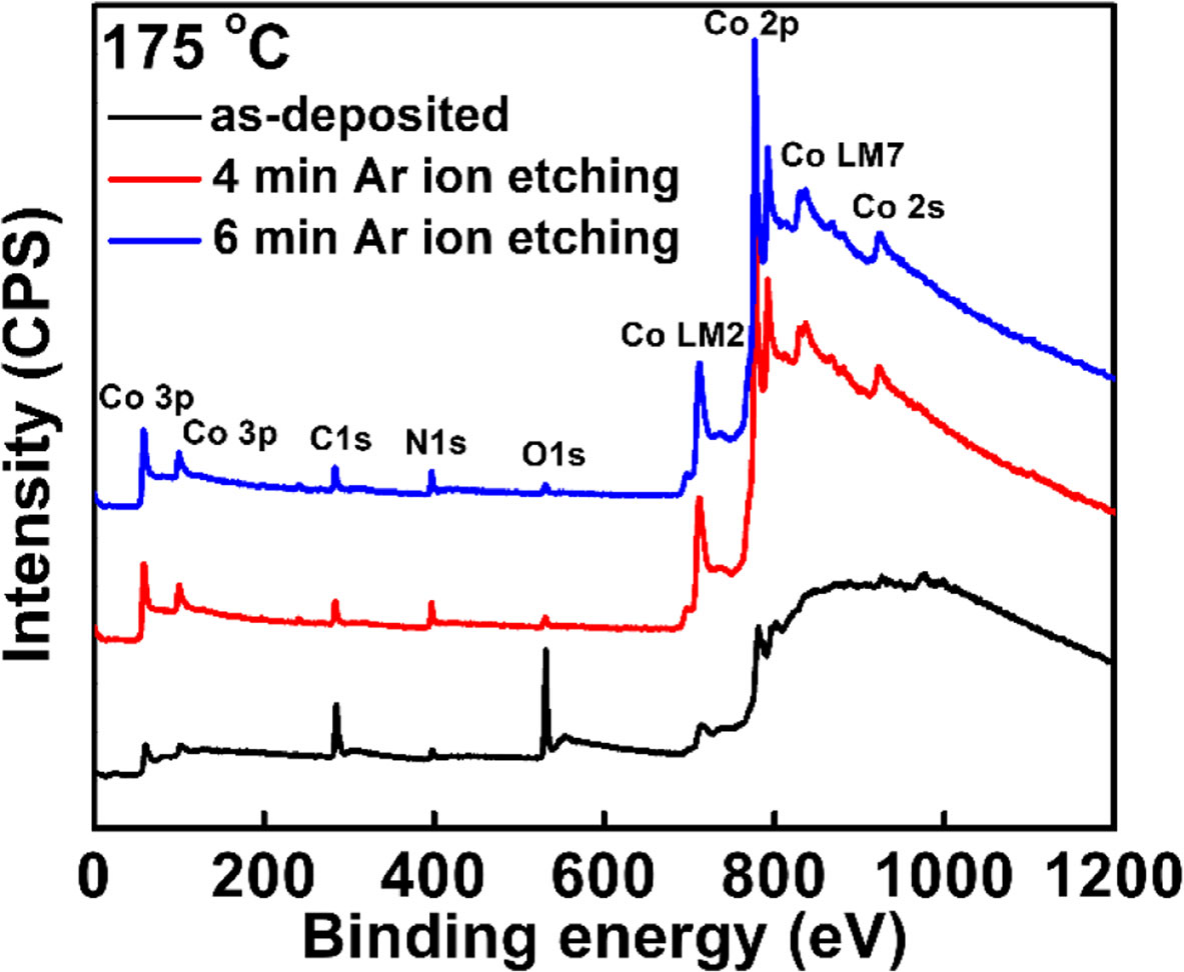
The survey spectra of the Co films deposited at 100 °C with different etching time

Figure 2 shows the survey XPS spectra of the films deposited at 175 °C. The deposited films consist of Co, N, O, and C elements. It is worthwhile to mention that to completely remove the surface contamination, all the samples were etched in situ with Ar ion bombardment for 6 min prior to the collection of XPS spectra. Table 1 lists the elemental percentages of the film deposited at 175 °C with the etching time and the C and O contents remain constant after 6 min of etching, indicating a complete removal of surface contamination. Table 2 lists the elemental atom ratio of the films grown at 100 and 175 °C, respectively, which are extracted from the high-resolution XPS spectra. As the substrate temperature increases from 100 to 175 °C, the relative content of C decreases from 40 to 32% and the elemental percentage of N increases from 14 to 18%. Moreover, the relative percentage of O shows a slight increment from 5 to 7%. The higher C contents should be ascribed to the part removal of the EtCp ligands [15, 16]. Since there is no O element in the precursors, the O atoms in the deposited films are probably originated from the oxygen in the reaction chamber.

**Table 1:** The element percentage in the Co films deposited at 175 °C with different etching time

175 °C	Co	C	N	O
Surface	10%	54%	6%	30%
4 min etching	46%	30%	19%	5%
6 min etching	45%	32%	18%	5%

**Table 2:** The element percentage in the Co films deposited at 100 °C and 175 °C, respectively after etching

After etching	Co	C	N	O
100 °C	39%	40%	14%	7%
175 °C	45%	32%	18%	5%

**Fig. 3:**
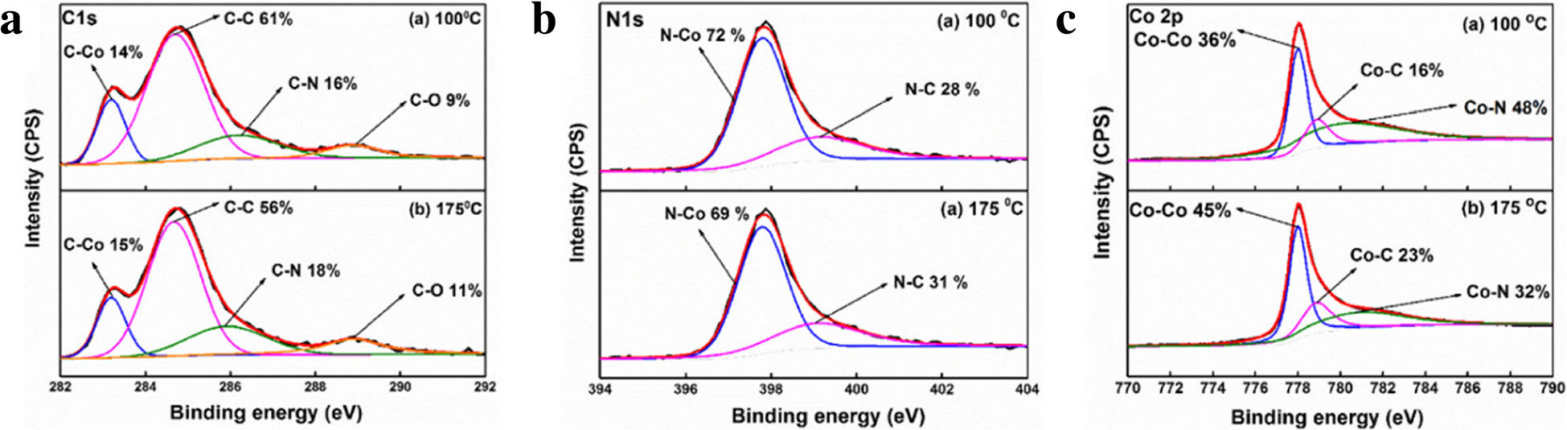
High-resolution **a** Co 2p_3/2_, **b** C 1s, and **c** N 1s XPS spectra of the films deposited at 100 °C and 175 °C, respectively

Figure 3a shows high-resolution C 1s spectra of the films deposited at different temperatures. The C 1s spectrum can be well divided into four components at 283.2, 284.7, 286.1 ± 0.1 eV, and 288.9 eV, which result from C-Co [12], C-C [12, 18], C-N [15, 19], and C-O [12] bonds, respectively. As the temperature increases from 100 to 175 °C, the relative content of C-C bond decreases from 61 to 56%, whereas those of C-Co, C-N, and C-O increase by 1%, 2%, and 2%, respectively. This reveals that more EtCp ligands were decomposed at a higher temperature, thus leading to the reduction of the relative percentage of C in the film. Figure 3b shows the high-resolution N 1s spectra of the films deposited at different temperatures. Each N 1s spectrum can be well separated into two components using the Gaussian-Lorentzian function. The peak located at 397.8 eV should be associated to N-Co bond [13], and the peaks centered at 399.2 eV should be corresponding to N-C [20, 21] bond. As the substrate temperature increases from 100 to 175 °C, the relative content of N-Co decreases from 72 to 69%. This is because the desorption of nitrogen from the film is enhanced at higher temperature, resulting in the formation of less N-Co bonds. Figure 3c shows high-resolution Co 2p_3/2_ XPS spectra of the Co films deposited at different temperatures. Regarding the existence of the C-Co and N-Co bonds, as revealed in Fig. 3a, b, it is reasonable that the Co 2p_3/2_ spectrum can be separated into three components, which are located at 778, 778.9, and 780.86 ± 0.34 eV, respectively. The peak at the smallest binding energy should be attributed to the Co-Co bond [12, 22]. Furthermore, since the Pauling electronegativity of C (2.55) is smaller than that of N (3.04), the positive charge density on Co bonded to N is larger than that on Co bonded to C. Therefore, the peaks at 778.9 and 780.86 ± 0.34 eV should arise from the Co-C and Co-N bonds, respectively. When the deposition temperature is increased from 100 to 175 °C, the relative content of Co-N bond decreases from 48 to 32%, which is consistent with the evolution of N-Co bond in Fig. 3b.

**Fig. 4:**
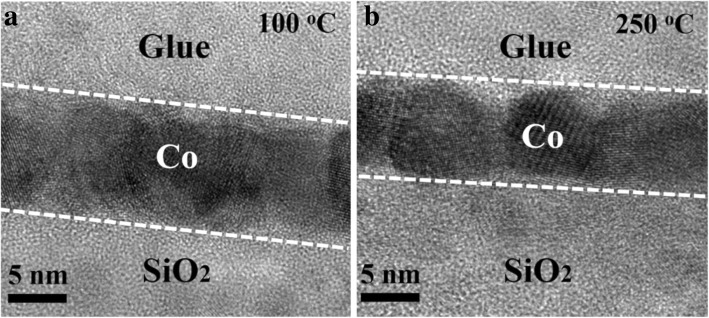
The cross-sectional TEM images of the Co films (1200 cycles) deposited at **a** 100 °C and **b** 250 °C, respectively

**Fig. 5:**
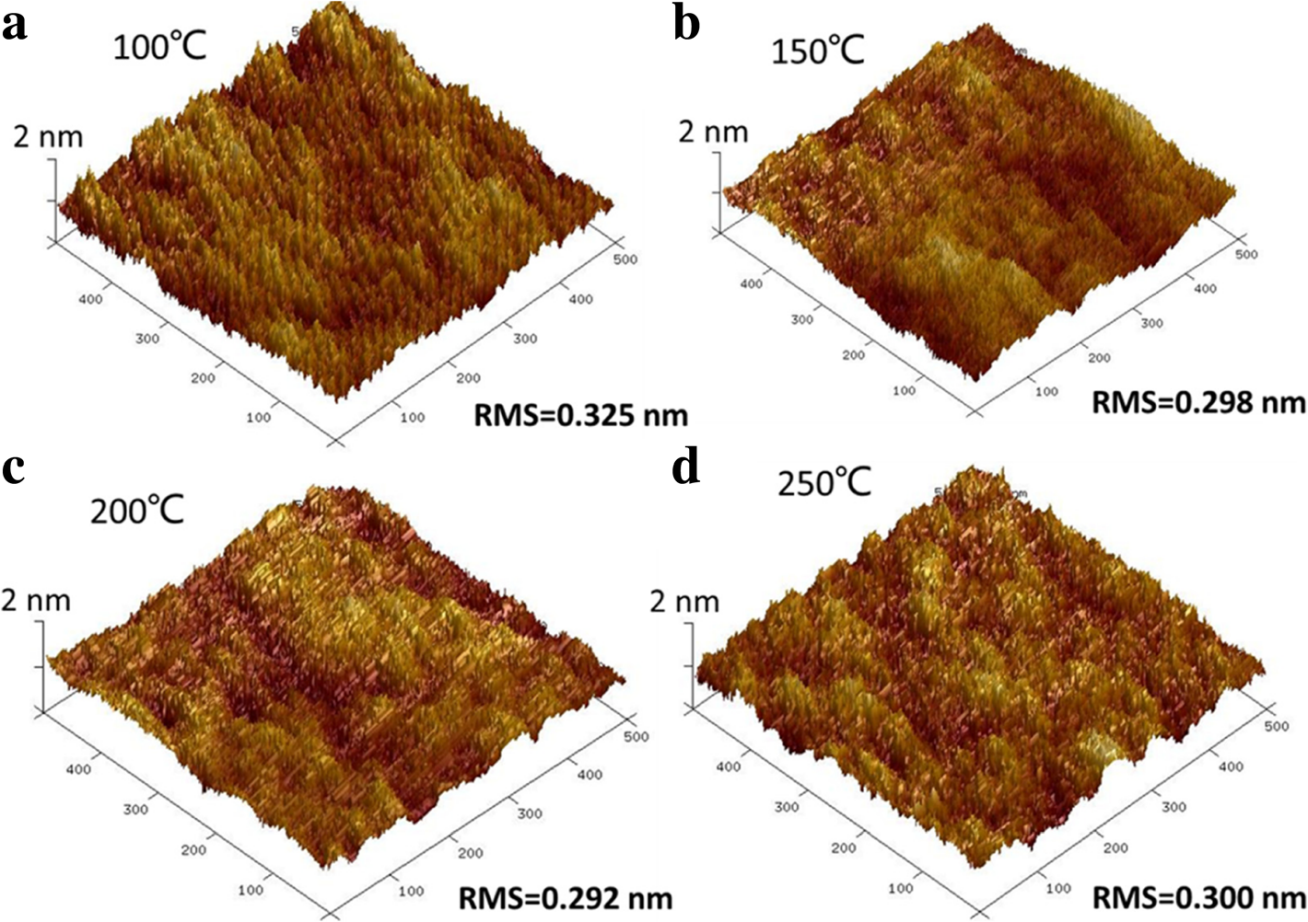
AFM images of the Co films (1200 cycles) deposited with Co(EtCp)_2_ pulse time of 2 s and NH_3_ plasma pulse time of 10 s at different temperatures: **a** 100 °C; **b** 150 °C; **c** 200 °C; **d** 250 °C

The crystal properties of the Co films are characterized by TEM, as shown in Fig. 4. The Co films deposited at 100 and 250 °C, respectively, are both crystallized. Figure 5 shows the AFM pictures of the Co films grown at different temperatures. As the deposition temperature increases from 100 to 250 °C, the root mean square (RMS) roughness shows a smaller variation and maintains a low value of ~ 0.3 nm, indicative of a flat Co film.

**Fig. 6:**
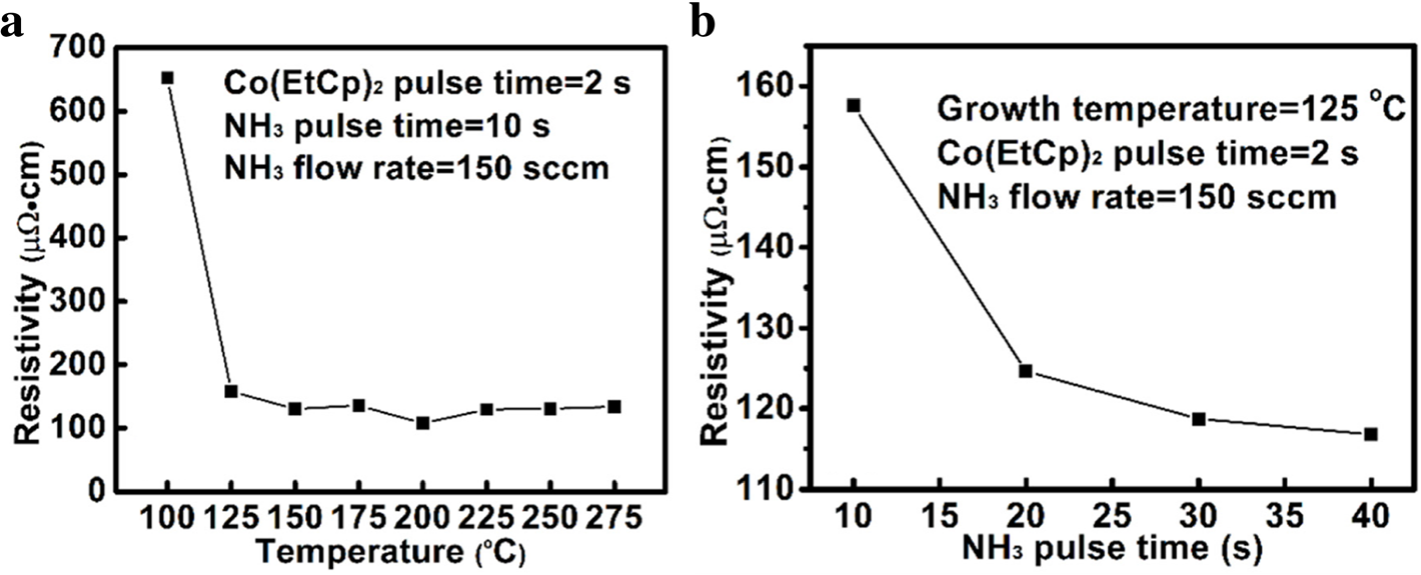
The resistivity of the Co film as a function of growth temperature (**a**) and NH_3_ pulse time (**b**), respectively

**Fig. 7:**
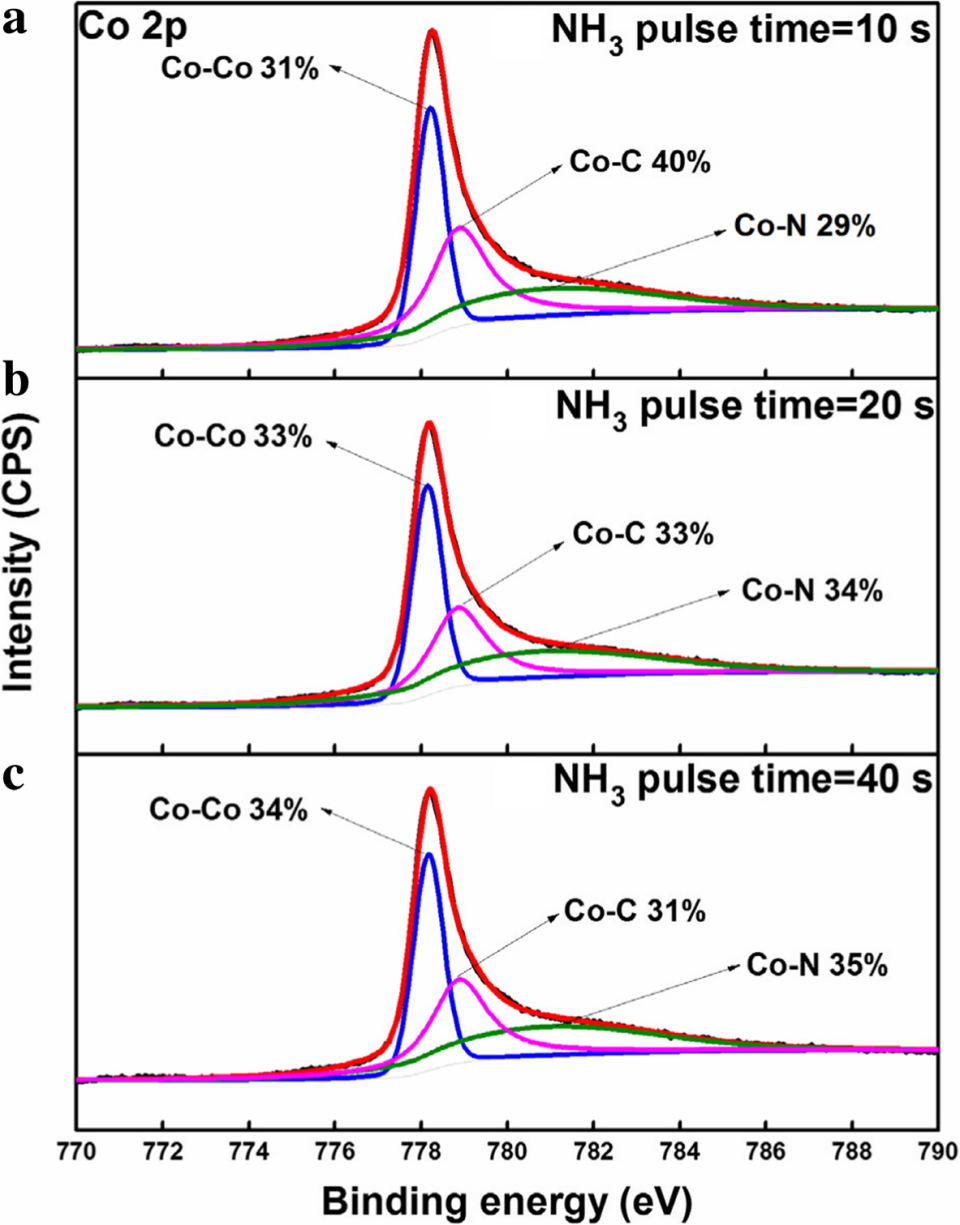
The XPS spectra of the Co films with different NH_3_ plasma times: **a** 10 s; **b** 20 s; **c** 40 s

Figure 6a, b shows the dependence of the resistivity of the Co films on the substrate temperature and NH_3_ pulse time. The film resistivity remarkably reduces from 652 to 130 Ω cm and then keeps constant with increasing the deposition temperature from 100 to 275 °C. The results should be attributed to the increase in the relative content of metallic Co in the film. As the NH_3_ pulse time increases from 10 to 40 s, the resistivity of the Co films decreases from 158 to 117 μΩ cm (see Fig. 6b). Figure 7 shows the Co 2p_3/2_ XPS spectra of the Co films deposited with different NH_3_ plasma times. As the NH_3_ plasma time is prolonged from 10 to 40 s, the relative percentage of the Co-Co bonds increases from 31 to 34%, and the relative content of the Co-C bonds decreases from 40 to 31%. Furthermore, the carbon content decreases by 22% as the NH_3_ plasma pulse time increases from 10 to 40 s. This indicates the gradually increased removal of the EtCp ligands, thus contributing to the decrease of the Co film resistivity.

**Fig. 8:**
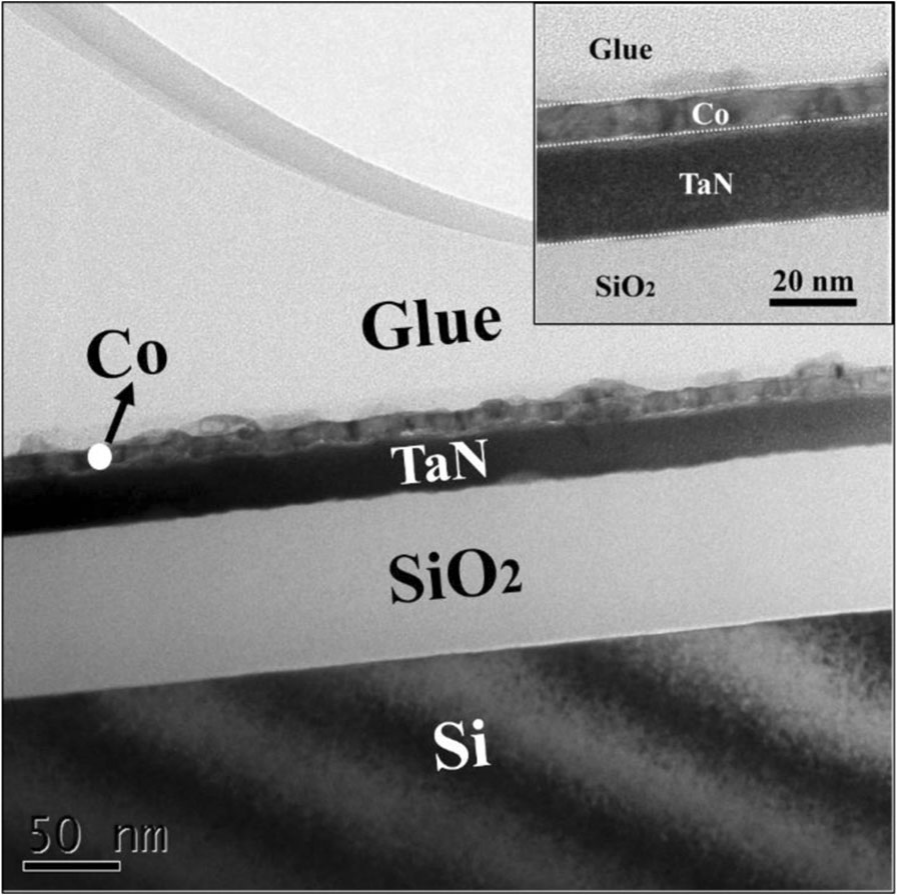
The cross-sectional TEM image of the Co film (1200 cycles) deposited at 125 °C on the ALD TaN surface and the inset is a magnified picture

Table 3 shows the performance comparison of the Co films grown by different precursors. Compared with CoCp_2_ and Co(MeCp)_2_, Co(EtCp)_2_ enables the atomic layer deposition of Co films at a lower temperature. In addition, using the metal precursor of Co(EtCp)_2_ leads to a smaller growth rate. Theoretically, the ALD process is the growth of monolayer by monolayer. In fact, the steric hindrance effect and surface adsorption of precursors both affect the film growth. In terms of the steric hindrance effect, it means that the ligands of the chemisorbed metal precursor species can shield partial surface, and prevent other metal precursor species from being adsorbed fully on the substrate surface. Since Co(EtCp)_2_ has a larger ligand compared with CoCp_2_ and Co(MeCp)_2_, it is assumed that a significant steric hindrance effect will occur during the ALD process. This can lead to the growth of sub-monolayer, thus resulting in a smaller growth rate. On the other hand, since a Co liner layer should be deposited on a TaN barrier layer for the future practical applications, 1200 cycles of Co films were grown on the ALD TaN film at 125 °C. Figure 8 shows the cross-sectional TEM image of the Co film deposited on the TaN surface. It is observed that the Co film is continuous and uniform, revealing a superior growth behavior. Further, it is found that the thickness (about 10 nm) of the Co film deposited on the ALD TaN film is similar to that grown on the SiO_2_ surface.

**Table 3:** The performance comparison of the Co films grown by different precursors

Precursors	Reactant gas	GPC (Å)	Process window (°C)	Resistivity (μΩ cm)	Ref.
CoCp_2_	NH_3_ plasma	0.48	250–400	< 20 @300 °C	[11]
Co(MeCp)_2_	NH_3_ plasma	0.4–0.6	200–350	30–400	[15]
Co(EtCp)_2_	NH_3_ plasma	0.12	125–225	129–158	This work

## Conclusions

The growth of Co thin film is devolved by PE-ALD using the precursors of Co(EtCp)_2_, and the influence of process parameters on the characteristics of the Co films were investigated. The addition of Et group to Cp ligand enables the process window moved to a lower deposition temperature of 125 °C. Moreover, the Co films are composed of Co and C element together with some N and O elements. With increasing the deposition temperature, the EtCp ligands are removed more sufficiently and the relative elemental percentage of C is decreased. As a consequence, the resistivity of the deposited Co films reduces from 652 to 130 μΩ cm and then remains a stable value when the substrate temperature is increased from 100 to 275 °C. For the deposition temperature of 125 °C, the resistivity is gradually decreased with the prolongation of NH_3_ pulse time and a low resistivity of 117 μΩ cm can be obtained when a NH_3_ pulse time of 40 s is used. The root mean square roughness shows a smaller variation with the deposition temperature and maintains a low value of ~ 0.3 nm, indicative of a flat Co film.

## Abbreviations

AFM: Atomic force microscopy
ALD: Atomic layer deposition
Co(CpAMD): Cyclopentadienyl isopropyl acetamidinato cobalt
Co(EtCp)_2_: Bis(ethylcyclopentadienyl) cobalt
Co(MeCp)_2_: Bis(η-methylcyclopentadienyl) cobalt
CoCp_2_: Bis-cyclopentadienyl cobalt
Cp: Cyclopentadienyl
ILD: Interlayer dielectric
PE: Plasma-enhanced
RMS: Root mean square
SEM: Scanning electron microscope
XPS: X-ray photoelectron spectroscopy
XRD: X-ray diffraction
